# Genotype-phenotype correlations of *GFAP* variants in type I Alexander disease subtypes

**DOI:** 10.1016/j.ymgme.2025.109689

**Published:** 2025-11-30

**Authors:** Tiziana Bachetti, Ylenia Vaia, Alice Grossi, Francesca Rosamilia, Enrico Bertini, Francesco Nicita, Deianira Bellitto, Florian Eichler, Geneviève Bernard, Amanda Nagy, Ayelet Zerem, Morteza Heidari, Ali Reza Tavasoli, Isabella Moroni, Isabella Ceccherini, Davide Tonduti, AxD Type I Study Group

**Affiliations:** a UO Proteomica e Spettrometria di Massa, IRCCS Ospedale Policlinico San Martino, Genova, Italy; b Unit of Pediatric Neurology, C.O.A.L.A. (Center for Diagnosis and Treatment of Leukodystrophies), V. Buzzi Children’s Hospital, Milan, Italy; c Neuroscience Research Center, Biomedical and Clinical Sciences Department, University of Milan, Milan, Italy; d UOSD Area Aggregazione Laboratori della Ricerca, IRCCS Istituto Giannina Gaslini, Genoa, Italy; e Clinical Bioinformatics, IRCCS Istituto Giannina Gaslini, Genoa, Italy; f Unit of Muscular and Neurodegenerative Diseases, Bambino Gesù Children’s Hospital, IRCCS, Rome, Italy; g Department of Neurology, Massachusetts General Hospital, Boston, MA, USA; h Harvard Medical School, Boston, MA, USA; i Departments of Neurology and Neurosurgery, Pediatrics, and Human Genetics, McGill University, Montreal, Quebec, Canada; j Child Health and Human Development Program, Research Institute of the McGill University Health Centre, Montreal, Canada; k Pediatric Neurology Institute, Dana-Dwek Children’s Hospital, Tel Aviv Sourasky Medical Center; Faculty of Medical and Health Sciences, Tel Aviv University, Tel Aviv, Israel; l Pediatric Neurology Division, Myelin Disorders Clinic, Children’s Medical Center, Pediatric Center of Excellence, Tehran University of Medical Sciences, Tehran, Iran; m Department of Neurology, Barrow Neurological Institute at Phoenix Children’s Hospital, AZ, USA; n Department of Pediatric Neurosciences, Fondazione IRCCS Istituto Neurologico Carlo Besta, Milan, Italy

**Keywords:** Gene variant, GFAP, Genotype-phenotype correlation, Neurodegeneration, Alexander disease

## Abstract

**Introduction:**

Alexander disease (AxD) is a rare and progressive leukodystrophy caused by variants in the Glial Fibrillary Acidic Protein (GFAP) gene. AxD presents as Type I and Type II, based on clinical and neuroradiological features. However, Type I patients variable disease progression has led to sub-classify them into subtypes Ia-Ib-Ic-Id. Our study investigated genotype-phenotype correlations in Type I AxD across these subtypes.

**Methods:**

A cohort of 74 genetically confirmed Type I AxD patients was analysed, with variants classified according to the clinical subtypes Ia, Ib, Ic, and Id. Genotypic–phenotypic correlations were explored both across all subtype groups and in stratified analyses based on patients’ ability to achieve independent ambulation versus those who never acquired this milestone. To investigate the functional consequences of a representative variant, in vitro assays were performed. Selected mutant GFAP protein was expressed and their ability to assemble into intermediate filament networks was assessed.

**Results:**

The study revealed a progressive decrease in GFAP allelic heterogeneity across disease subtypes from Ia to Id. Additionally, *GFAP* pathogenic variants in exon 1 were more frequent in milder forms, whereas mutations in exon 4 were predominantly observed in severe phenotypes. In addition, changes at p.R239 and p.R79 residues showed an opposite trend, with variants affecting p.R239 never detected in Id subtype, while variants affecting p.R79 never detected in Ia. Specific aminoacidic changes at the p.R79 residue were able to stratify patients based on ability to acquire independent ambulation or not. Furthermore, the distribution of Combined Annotation Dependent Depletion (CADD) scores showed an inverse correlation with the acquisition of autonomous ambulation.

**Conclusions:**

These results suggest that specific *GFAP* variants could be potential predictors for disease progression in Type I AxD, supporting patients’ subclassification and underscoring the importance of considering both genetic and clinical factors in AxD management, particularly as therapeutic interventions are being developed.

## 1. Introduction

Alexander disease (AxD) is a rare, progressive leukodystrophy caused by autosomal dominant gain-of-function pathogenic variants in the Glial Fibrillary Acidic Protein (*GFAP*) gene, which encodes GFAP, the primary intermediate filament protein in astrocytes [1,2]. These variants lead to the formation of GFAP oligomers called Rosenthal Fibers (RFs), which negatively impact myelin by disrupting various intracellular mechanisms [3–5].

Clinically, AxD entails a wide phenotypic spectrum, and several classifications are currently in use, with the first classification based on age of onset [6,7], and subsequent taking into account radiological features and neurological findings at onset or during the disease course [8,9]. In particular, Prust et al. identified a Type I AxD characterized by early onset (usually less than 4 years), “classic” neuroradiologic features, seizures, macrocephaly, and developmental delay and a Type II AxD, manifesting across the lifespan, and characterized by “atypical” radiologic features, mainly bulbar symptoms and autonomic dysfunction [8]. However, patients classified according to this model may exhibit significantly different disease progression [10,11]. To account for variations in disease progression among Type I AxD patients, a subclassification system was introduced, categorizing patients into subtypes Ia, Ib, Ic, and Id based on their disease course over time [10,11]. Patients defined as Type Ia AxD show a very severe clinical course from onset, with no acquisition of gross motor milestones, rapidly evolving course, and death by the second year of life. Type Ib includes patients who never achieve autonomous ambulation and who begin to deteriorate before the age of 6 years, with a rapid loss of previously acquired skills. Diversely, Type Ic patients present a delay in neuromotor development, but then achieve autonomous ambulation; their clinical course appears to be initially stable and clinical deterioration only manifests between 6 and 12 years of age, eventually leading to a loss of previously acquired skills. Finally, Type Id patients may present a delay in neuromotor development, but acquire autonomous ambulation, eventually showing intellectual disability. Their clinical course is slower, with apparent clinical stability beyond 12 years. Patients that had acquired autonomous ambulation but were too young to fit in either subtype Ic or Id were defined as Ic/Id [10,11]. The underlying causes of such phenotypic variability are unknown yet.

Since pathogenic variants in *GFAP* were identified as the cause of AxD in 2001 [12], many *GFAP* variants have been reported to be associated with the disease [13]. A genotype-phenotype correlation has been reported for the two most frequently mutated arginine (Arg, R) residues (p.R79 and p.R239), with the phenotype associated with variants affecting p.R79 apparently less severe than that associated with variants affecting the p.R239 residue, but no broader phenotypic pattern has been defined [14]. Three sets of identical twins have been reported showing extraordinarily similar disease courses, additionally suggesting that genetic factors may play a significant role [15–17]. These findings support the hypothesis that phenotypic variability in AxD may be influenced by genetic modifiers. In 2019, Yasuda et al. attempted to identify the genetic variations responsible for modifying the disease phenotype, with no genome-wide significant variant identified, but recognizing several variants that appear likely related to the age at disease onset [18].

A recent systematic review of 550 *GFAP* variants identified in AxD documented the distribution of the most common *GFAP* variants involving Arg residues across all categories of onset, identifying correlations between Combined Annotation Dependent Depletion (CADD), a pathogenicity score parameter, and age of onset, inheritance patterns, and GFAP protein domains, thus suggesting a potentially predictive role of genetic factors in AxD outcomes [13]. Moreover, during the preparation of this manuscript, a very recent study was published reporting correlations between GFAP variants and patient survival in both early- and late-onset disease [19].

Recent advances in diagnostics and drug development have facilitated the identification of therapeutic candidates for AxD. Notably, disease-modifying therapies are being explored, including antisense oligonucleotide treatment currently in clinical trials (NCT04849741) [20].

In this scenario, the identification of suitable disease modulators that are predictive of the disease phenotype is crucial [21].

This work aims to deepen our knowledge on the genotype-phenotype correlation in AxD, to provide further useful information for the prognosis of Type I AxD.

## 2. Methods

### 2.1. Patients’ enrolment

Patients with confirmed clinical and molecular diagnosis of AxD were enrolled worldwide. Data were extracted from medical record and collected via RedCap tool [22,23] at each institution and centralized at V. Buzzi Children’s Hospital. Retrospective fine-grained data collection was performed to include demographic information, age at disease onset, DNA variants, cognitive and motor development, age of independent ambulation acquisition, and other relevant clinical information regarding disease evolution over time, including age of onset of neurological decline and age of possible loss of independent ambulation. The age of onset of neurological decline was defined as the time point at which previously acquired motor and/or non-motor skills were lost, to allow application of the subclassification method. [11] Based on available clinical information, Type I AxD patients were subclassified according to disease evolution over time. In addition, for refinement of analysis, a cohort of Type I AxD patients selected from the literature review published by Vaia et al. in 2023 was included [11]. Furthermore, an additional review of papers published between 2023 and 2024 was performed, including case series and case reports of subjects with AxD, and new patients from the literature that were described reporting enough clinical and genetic information for subclassification were included.

For any variable, patients with relevant and complete data were included in each statistical analysis. The age at onset and changes in independent ambulation (acquisition or loss) were recorded in months rather than years, to allow for more precise detection of differences.

### 2.2. GFAP variants pathogenicity prediction tools

Two metrics were applied to assess the potential pathogenicity of the identified variants: the Combined Annotation Dependent Depletion (CADD) score and CADD-Splice (CADD v1.6; updated on 31st December 2023) [24]. CADD integrates diverse genomic and epigenomic features, including sequence context, gene annotations, evolutionary conservation, and functional predictions, to estimate the relative deleteriousness of single nucleotide and small insertion/deletion variants.

The CADD score represents a relative ranking rather than an absolute prediction. Higher values indicate variants more likely to have a functional impact. While no fixed cutoff is defined, scores above 20 generally correspond to variants within the top 1 % of the most deleterious possible substitutions in the human genome [24].

### 2.3. Transient transfection of GFAP expression plasmids and fluorescence microscope analysis

The plasmids pcDNA3.1CT-GFP-p.R239H and pcDNA3.1CT-GFP-p. R79H GFAP were constructed through mutagenesis (GenScript) from the previously generated pcDNA3.1CT-GFP-TOPO-GFAP WT template and transfected in Human U251-MG astrocytoma cells as previously described [25].

Human U251-MG astrocytoma cells were grown in RPMI supplemented with Fetal Bovine Serum 10 %, Penicillin, Glutamine, Streptomycin. Fifty thousand cells were transiently transfected in 4 wells chamber slides (Life Technologies) with Lipofectamine 2000 (Life Technologies) and 250 ng p.R239H-GFP or p.R79H-GFP expression plasmids (3.1 ratio); after 48 h, cells underwent fixation in paraformaldehyde 4 %/PBS (Sigma-Aldrich), followed by nuclei staining with DAPI (Life Technologies) and ProLong^™^ Diamond Antifade Mountant addition (Life Technologies); analyses were performed by fluorescence microscopy with 40× magnification (Zeiss, Axiophot) equipped with a Nikon camera. Images were acquired with NIS-Element software and merged by ImageJ imaging software. For each construct, at least 30 cells were analysed for GFAP conformation.

### 2.4. Statistical analysis

The distribution of the variants among the selected patients was expressed as percentages and was described with pie charts and bar diagrams by Office 2019 Excel and SRPlot [26]. The Shapiro-Wilk Test Calculator [27] was used to statistically evaluate the distribution of data. For non-normal distributions, the Wilconxon test non-parametrical was used, with significant threshold *p*-value <0.05. For correlation studies, linear regression analysis was performed, and the regression coefficient (R [2]) was calculated. For fluorescence microscopy analysis, a paired Student *t*-test was applied between comparisons; values were the mean of four independent experiments ±SD. Statistical significance was considered for p-value<0.05. Outliers were identified by GraphPad [28].

## 3. Results

### 3.1. Demographics

Forty-two pediatric patients with a clinical and genetic diagnosis of AxD were collected by 7 centers worldwide. Three patients were classified as type Ia, 11 as type Ib, 11 as type Ic, 9 as type Ic/Id, and 8 as type Id. In addition, 65 patients previously selected and classified from the literature [11] were added; of these patients, 19 were classified as type II by the authors, and thus excluded from our study. Of the remaining 46, 13 had a diagnosis based on neuropathological findings, and no pathogenic variants associated, and were excluded as well. Two patients had a non-missense pathogenic variant and were excluded from CADD analysis. The remaining 31 were considered eligible for inclusion, and their classification based on subtypes was considered, consisting in 10 type Ia, 10 type Ib, 5 type Ic, 3 type Ic/d, 3 type Id. (Table 1). In addition, one case published very recently and with sufficient clinical information was included and classified as type Ia [29], for a total of 32 patients from the literature (Table 1). The final cohort included 74 patients: 14 Ia, 21 Ib, 16 Ic, 12 Ic/d, and 11 Id.

### 3.2. Correlation between age of onset and Type I subtypes

The earliest classification of AxD, which stratifies patients based on age of onset, was examined in this study [6]. We investigated whether variations in age of onset corresponded to differences in the clinical phenotype of AxD Type I subtypes.

We observed a progressive increase in the median values of age of onset from subtypes Ia to Id [Ia = 0.6 months (range 0–2), Ib = 4.8 months (range 0,96–18), Ic = 9.5 months (range 1.33–24), Ic/d = 12.2 months (range 3.6–21.96) and Id = 19 months (range 9.6–24)] (Fig. 1A) with a high level of correlation, (regression coefficient R^2^ = 0.9198) (Suppl. Fig. 1). In particular, most two-by-two comparisons showed statistically significance (Ia vs Ib: *p* = 3.9 × 10^−6^; Ia vs Ic: *p* = 1.3 × 10^−5^; Ia vs Ic/Id: *p* = 2 × 10^−5^; Ia vs Id: *p* = 0.00017; Ib vs Ic: *p* = 0.014; Ib vs Ic/Id: *p* = 0.0025; Ib vs Id: *p* = 0.0034).

In addition, the distribution of the age of onset showed statistically significant higher mean and median values in the subtypes that were able to acquire autonomous ambulation (Ic, Ic/Id, Id) (*p* = 0.011) (Fig. 1B). However, although the age of onset in groups Ia and Id shows little variability and no overlap, the high variability observed in the Ic and Ic/Id groups prevents the use of age of onset alone as a reliable predictor of outcome for an individual child.

Frequency of affected *GFAP* exons in the Type I AxD subtypes.

To explore possible associations between subtypes and *GFAP* exon involvement, we analysed the frequency of affected exons in each AxD Type I subtype. *GFAP* variants in exons 1, 2, 4, 6, 7, and 8 were represented at a different extent in each disease subtype (Fig. 2). Exon 4 was involved at variable degree in all subtypes except Id, while exon 1 showed a progressive increased involvement from Ia to Id (Suppl. Fig. 2). Moreover, a marked decrease in GFAP variants affecting exons 4 and 6 was observed across subtypes from Ia to Id (Fig. 2). Similarly to age of onset, also exon parameter alone cannot be considered truly predictive; however, the exon 1-Id association is highly suggestive of a milder outcome, that, if combined with other scores, could be used as predicting factor.

### 3.3. GFAP variants distribution in Type I AxD subtypes

We then focused on the specific variants identified in AxD Type Ia, Ib, Ic, Ic/Id, and Id patients. The analysis revealed a non-uniform distribution of *GFAP* variants across AxD Type I subtypes (Fig. 3), highlighting the need for further investigation. In particular, a quality analysis was performed to evaluate exclusive and common variants between groups. A preliminary observation highlighted a progressive decrease in *GFAP* allelic heterogeneity of exclusive variants from Ia to Id, mainly represented by private *GFAP* variants. Notably, we detected 10 such variants in Ia, 7 in Ib, 3 in Ic, 3 in Ic/d and only 1 *GFAP* exclusive change in Id.

Moreover, although 24 variants were unique to specific subtypes, only 7 *GFAP* changes were shared among two or more subtypes. In particular, p.R239H and p.Y366C were present in Ia and Ib, p.V87I in Ic and Id, p.R239C in Ib, Ic, and Ic/d and, finally, p.R88C, p.R79C, and p. R79H in all subtypes except for Ia. Finally, it was observed that frequent substitutions affecting Arg residues were shared by all type I subtypes. Of note, a progressive increase in Arg to Cysteine (Cys, C) changes from Ia to Id was observed (R^2^ = 0.9402) (Suppl. Fig. 3).

### 3.4. Arginine residues among GFAP variants

In our cohort, R79, R88, and R239 were the codons showing the most frequent changes affecting Arg (R) residues; R79 (*n* = 25, 32 % of total variants) and R239 (*n* = 16, 22.6 % of total variants), R88 (*n* = 7, 9,4 %).

In particular, except for two cases (R79L and R88S), R79 and R239 were changed in Cys (C) or His (H), while R88 in Cys (C) only. Thus, considering the distribution of the specific changes in each AxD Type I subtype, Ia showed only the R239H change, Ib showed all Arg changes (R79C, R79H, R239C, R239H, R88C), Ic and Ic/d all but R239H, and Id only the two R79 changes (Fig. 4). Of note, R239H was identified only in Ia and Ib, thus never associated with Ic-Ic/d-Id subtypes.

### 3.5. Different contribution of arginine changes in acquisition of independent ambulation

After observing the different distribution of Arg residues among type I subtypes, we investigated the representation of alternative missense changes of the same Arg residues from Ia to Id, exploring whether some *GFAP* variants could stratify patients based on the acquisition of independent ambulation (Ic + Ic/d + Id) or not (Ia + Ib). Considering patients that never acquired independent ambulation, we observed a high presence of non-frequently changed Arg variants (20/35 “others” = 57,3 %) (Fig. 5A) while, among the frequently changed Arg variants (R239, R79 and R88), the R239 was the most frequently changed (9/35 = 25.71 %), followed by the R79 (5/35 = 14.28 %) (Fig. 5A).

The same analysis performed in patients who achieved independent ambulation revealed a marked reduction in the contribution of variants other than R79C, R79H, R239C, R239H, and R88C (“others”), together with a predominant contribution of R79C and R79H (Fig. 5B). Notably, the contribution of R239H disappeared in this subgroup. In addition, among patients who acquired independent ambulation and retained it (Ic/d and Id), we further examined the age at which independent ambulation was achieved, focusing on the most frequent Arg-substituting variants in these groups, R79H and R79C.A near-significant difference (*p* = 0.05) was observed with the R79H variant tended to acquire walking ability earlier than those with R79C (Fig. 5C).

### 3.6. The R79H and R239H pathogenic variants induce different extent in aberrant GFAP assembly

To determine whether differences in the distribution of p.R239 and p.R79 variants might reflect specific GFAP conformational features, we investigated the ability of the GFAP p.R239H and p.R79H mutant proteins to properly assemble into the cytoskeletal network. Transient transfection of U251-MG astrocytoma cells, which endogenously express wild-type GFAP, with expression plasmids encoding GFP-fused GFAP p. R79H and p.R239H variants, to mimic a heterozygous condition, revealed a high proportion of cells containing aggregates, either alone or coexisting with filamentous structures (Fig. 6A). While mixed filament-aggregate forms appeared at similar frequencies in both mutants, p. R239H exhibited a higher proportion of cells with only aggregates. In addition, in these cell lines, where wild-type GFAP normally assembles correctly into filaments in 70–75 % of transfected cells [25], both mutant GFAP proteins exhibited a reduced ability to form proper filaments, as expected for GFAP variants associated with Type I AxD [20]. In particular, while p.R79H was able to produce filamentous patterns in near 20 % of cells, the p.R239H mutant could not correctly assemble in most cells (filaments R239H vs filaments R79H: *p* = 0.041), thus suggesting that the higher proneness to wrongly assemble could at least partially account for the more severe p.R239H associated clinical phenotype (Fig. 6B).

### 3.7. Distribution of the CADD score within the Type I subtypes

CADD score is a widely used measure of variant deleteriousness, due to single nucleotides or short indel changes [24,30], and was reported to show a different distribution between Type I and Type II AxD patients [13]. Due to these previous observations, we investigated whether this measure of pathogenicity could be useful to stratify Type I AxD patients.

CADD scores were significantly lower in the Id group compared to all other subtypes (Id vs Ia: *p* = 0.032; Id vs Ib: *p* = 0.027; Id vs Ic: *p* = 0.048; Id vs Ic/Id: *p* = 0.023) (Fig. 7A), whereas no significant differences were observed among the remaining subtypes. Since some CADD values in the Ia subtype were lower than expected, based on other values in the same group (T396I and p.N77S, CADD scores 22.5 and 25.6, respectively), we wondered whether CADD could be sensitive to some classes of variants. In particular, in silico phosphorylation for the two variants was predicted via NetPhos 2.0, confirming that p.T396I and p.N77S could alter GFAP phosphorylation, by loss and acquisition respectively (Suppl. Table 1) [31]. Since post-translational modifications are not among variables contributing to CADD score severity, these two phospho-variants were excluded from the analysis, leading to a marked decrease in the *p*-value of subtype Ia compared to Ib-Ic-Ic/d, although still not reaching a statistical significance (Fig. 7B). Given that inherited variants are generally linked to milder phenotypes compared to de novo variants, and that p.V115I in the Ia subtype was a statistical outlier (CADD = 15.2), we conducted an additional analysis excluding both phospho-variants (p.T396I and p.N77S) and inherited variants (p.V115F and p.V115I). This refined analysis revealed statistically significant differences in CADD score distribution (Fig. 7C). In particular, in addition to the statistically significant values already observed between Id and all single subgroups, elimination of the phospho-variants allowed to reach significant values also for Ia vs Ib (*p* = 0.023) and Ia vs Ic (*p* = 0.03).

Finally, by categorizing the CADD scores into three different severity ranges 20–27, 27–30, and 30–33, we observed an inverse correlation between the acquisition of independent ambulation and the CADD values (Suppl. Fig. 4).

## 4. Discussion

Type I AxD presents with a broad spectrum of disease trajectories, despite a more homogenous pattern of presentation at onset. The recently proposed subclassification (Ia–Id) seems to offer a useful framework for describing this heterogeneity [11]. In our cohort, disease evolution appeared broadly consistent with this model, with age at onset following the expected gradient from the most to the least severe forms. However, to date, this classification system may only be applied retrospectively and there are no existing markers able to predict disease evolution over time. Therefore, the present study explored whether a detailed analysis of *GFAP* variants could provide additional valuable prognostic insights and eventual correlations with Type I disease subtypes.

In particular, when considering the genetic background, our observations suggest that the position and nature of *GFAP* variants may influence disease expression. Variants located in exon 1 were generally found in patients with milder disease, while changes involving the C-terminal region tended to occur in those with more severe forms. This pattern is in keeping with previous hypotheses that structural alterations in the tail domain can compromise filament stability and astrocyte viability, leading to caspase-3 mediated astrocyte cell death [32]. Although causality cannot be firmly established, this domain-related difference may help explain some of the observed clinical variability.

Overall, most of the *GFAP* variants were exclusive to distinct disease subtypes, with allelic heterogeneity decreasing from Ia to Id, and variants at three Arg residues (p.R79, p.R88, p.R239) were the most common. In our cohort, patients carrying changes at R79, particularly R79H, often showed a relatively slower or less severe disease course compared to those with R239 substitutions, confirming what already reported in literature, with variants at residues R79 and R239 representing the different ends of the phenotypic spectrum [14]. The diverse clinical expressivity of variants such as p.R239H and p.R79H, may be attributed to the varying degrees of filamentous organization observed in cells transfected with expression plasmids encoding the two mutant GFAP proteins, showing that R79H may still allow partial filament formation, whereas R239H mainly produces aggregates. This may explain why the p.R239H variant exhibits complete penetrance, making it less susceptible to genetic background effects, as evidenced by its exclusive detection in infantile and neonatal cases. A very recent analysis of *GFAP* variants in AxD supports these findings, reporting the R79H variant in association with milder disease phenotypes, in terms of both age of onset and survival, compared with the R239H variant [19]. Moreover, p.R239H had been already reported in association with an earlier death, in a genetically heterogeneous cohort [11].

The contribution of post-translational mechanisms (i.e., citrullination, lipoxidation, ubiquitination) also deserves consideration. Variants affecting lysine residues (E373K, E312K), observed only in the most severe forms (Ia + Ib), might interfere with processes such as ubiquitination or other protein quality-control pathways [27]. Likewise, the apparent relationship between lower CADD scores and milder clinical forms could suggest a gradient of molecular disruption, but these scores should be interpreted with caution given their limited accuracy for complex or regulatory variants [33].

It is also possible that gene expression and background genetic variation modulate the phenotype. For example, the promoter SNP −250 A/C has been linked to differences in *GFAP* expression and may partly explain interindividual variability, even among patients with the same pathogenic variant,^36,37^ and *in trans* common variants may ascribe for phenotype variability found within each group, too [32].

This study has several limitations. The cohort size was small, reflecting the rarity of AxD and reducing the statistical power of the study. CADD score also suffers from some limitations. In fact, when it comes to autosomal dominant conditions, it is quite reliable for missense variants leading to pathogenetic gain-of-function aminoacidic changes, with structural effect on protein conformation, but it is less effective with variants related to other genetic processes, such as microdeletions/microinsertions that could be ascribed to splicing defects or frameshifts [33]. This led us to analyse CADD scores excluding variants predicted to alter phosphorylation sites, and inherited variants with reduced penetrance and/or variable expressivity. In addition, only the GFAP-α isoform was examined, leaving potential effects of other isoforms unaddressed.

Ultimately, it remains difficult to determine how much of the variability reflects the specific variants themselves and how much derives from additional modifying factors. Larger, multicentre studies will be needed to test the reproducibility of these observations and studies exploring additional modifying factors should be carried out.

In conclusion, our findings suggest that certain genetic features of *GFAP*, particularly the location of variants and their biochemical characteristics, may relate to disease severity in Type I AxD. Variants in exon 1, including changes at residue R79, were more often seen in milder subtypes, whereas substitutions affecting the C-terminal region, such as R239H, tended to occur in more severe cases (Fig. 8). While these associations are not deterministic, they may provide useful clues for early prognostic assessment when considered alongside clinical and imaging data. Continued collection of well-characterized cases and functional studies will be important to clarify how specific *GFAP* alterations shape the disease course.

## Supplementary Material

Supplementary data

## Figures and Tables

**Fig. 1.**
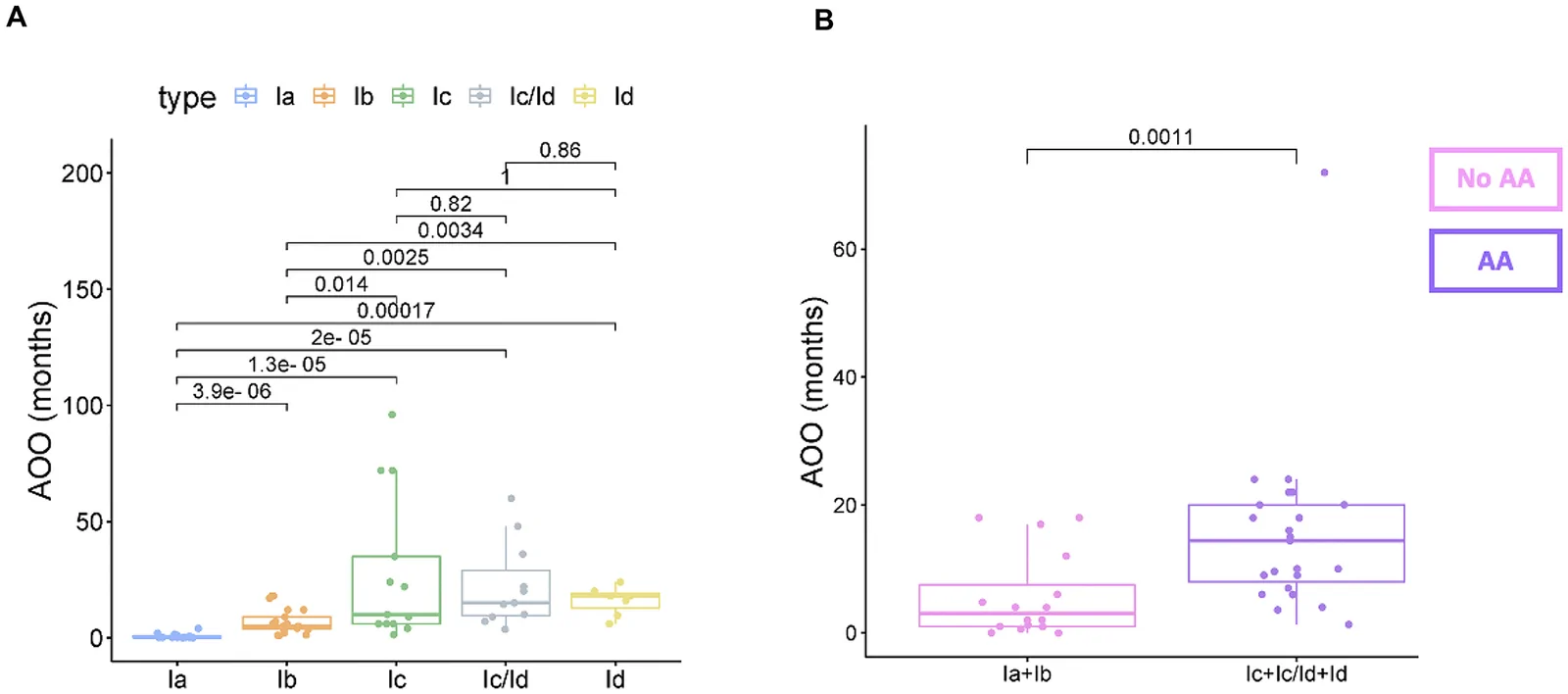
Age of onset in Alexander disease Type I subtypes Box plot showing the distribution of AOO expressed in months in each AxD type I subtype. Wilcoxson test *p*-value is indicated on the comparison bars. Anova multi-groups comparison p-value = 0.00015. Box plot showing the distribution of AOO median values, expressed in months, in type I AxD patients grouped based on the ability (Ic, Ic/d, Id) or not (Ia + Ib) to acquire AA. Student *t*-test p-value = 0.0058

**Fig. 2.**
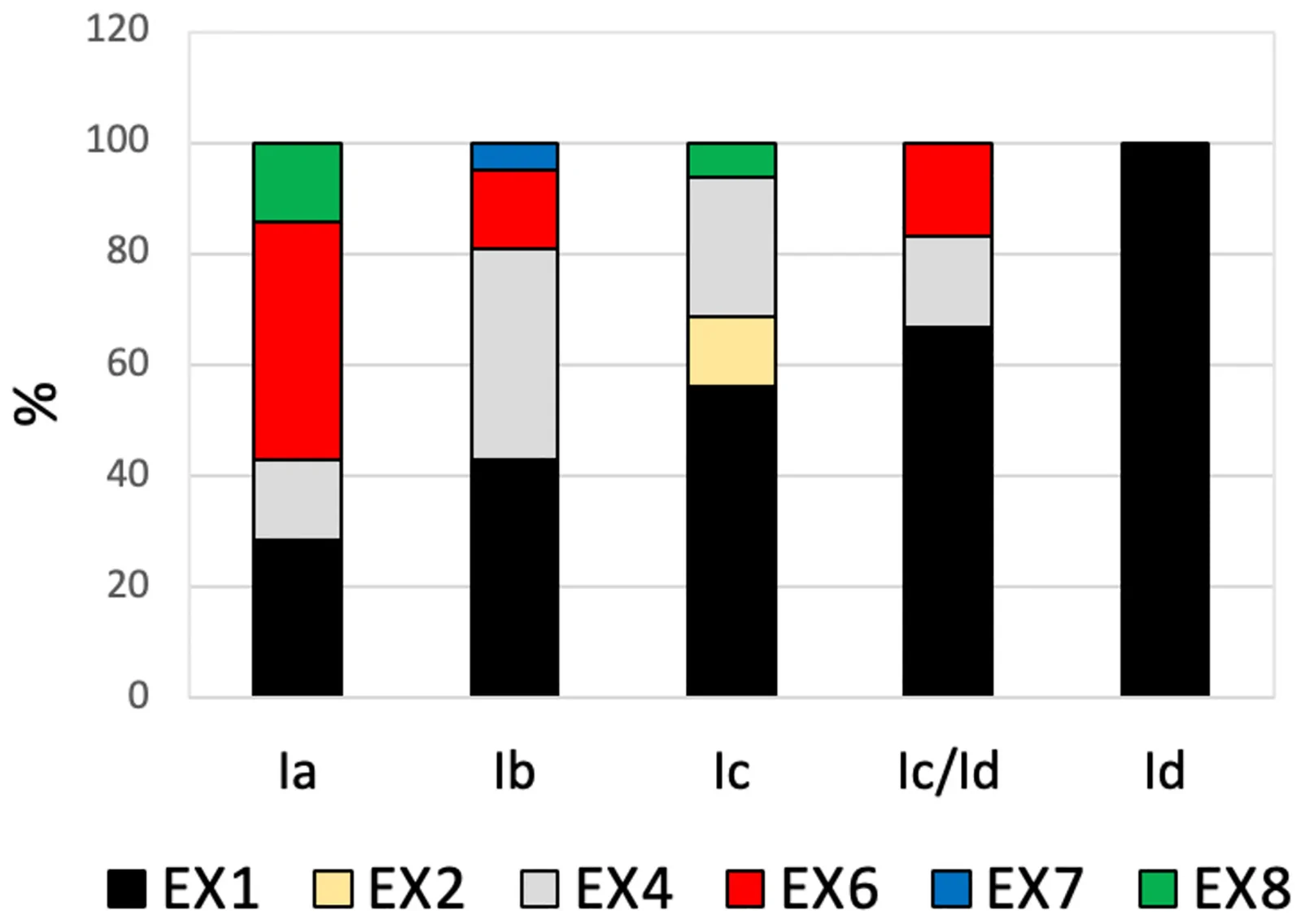
Distribution of *GFAP* exons in AxD Type I subtype. The bar diagram shows the contribution, expressed as a percentage, of variants in exons 1, 2, 4, 6, 7, and 8 in each Type I subtype. The true dimension of the coloured boxes represents the percentage value with respect to variants in all exons for each disease subtype (100 %). Abbreviations: EX = exon.

**Fig. 3.**
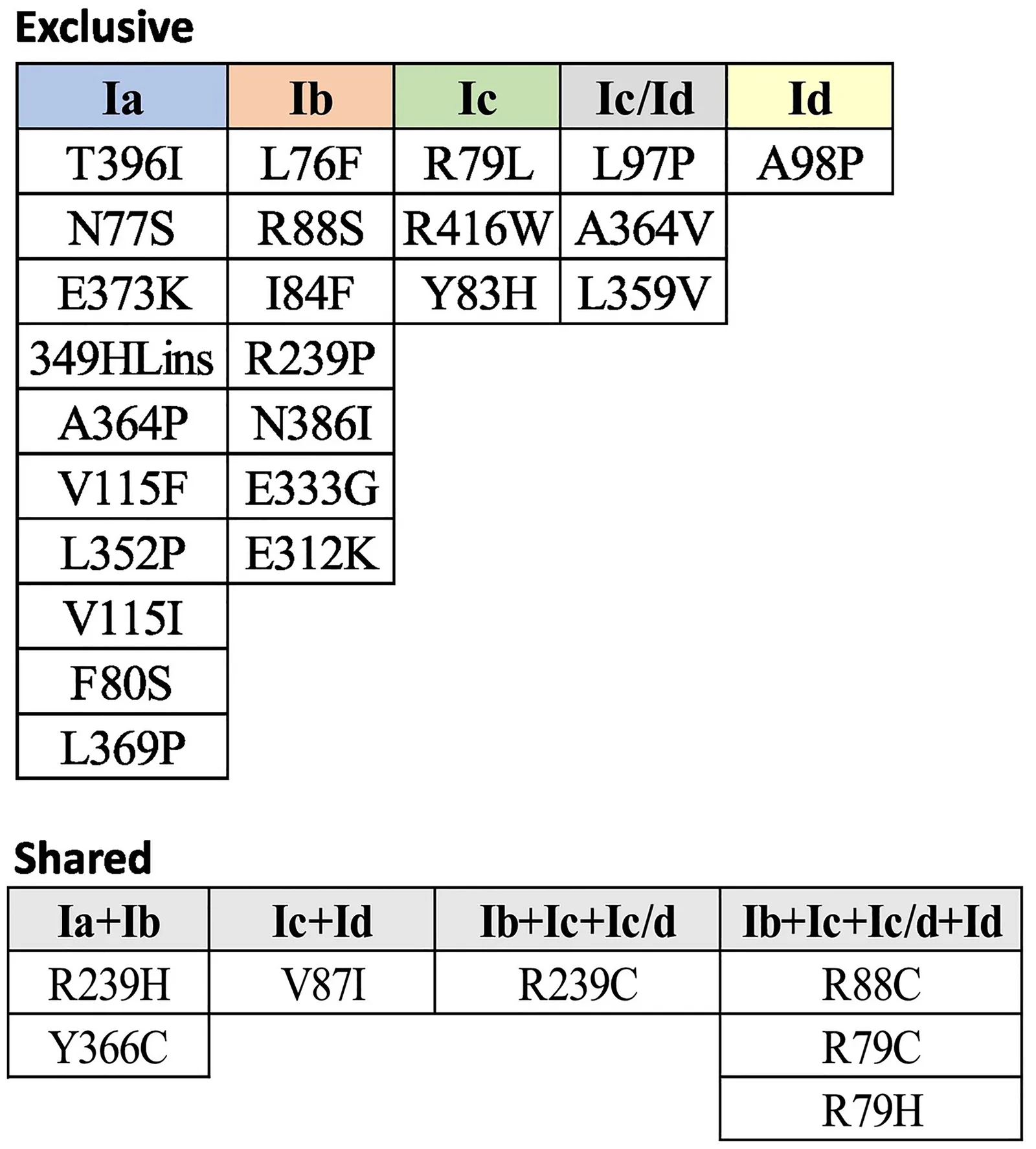
Distribution of *GFAP* variants in AxD Type I subtype. List of *GFAP* variants exclusive for each disease subtype (“unique to each group”) and shared between two or more groups.

**Fig. 4.**
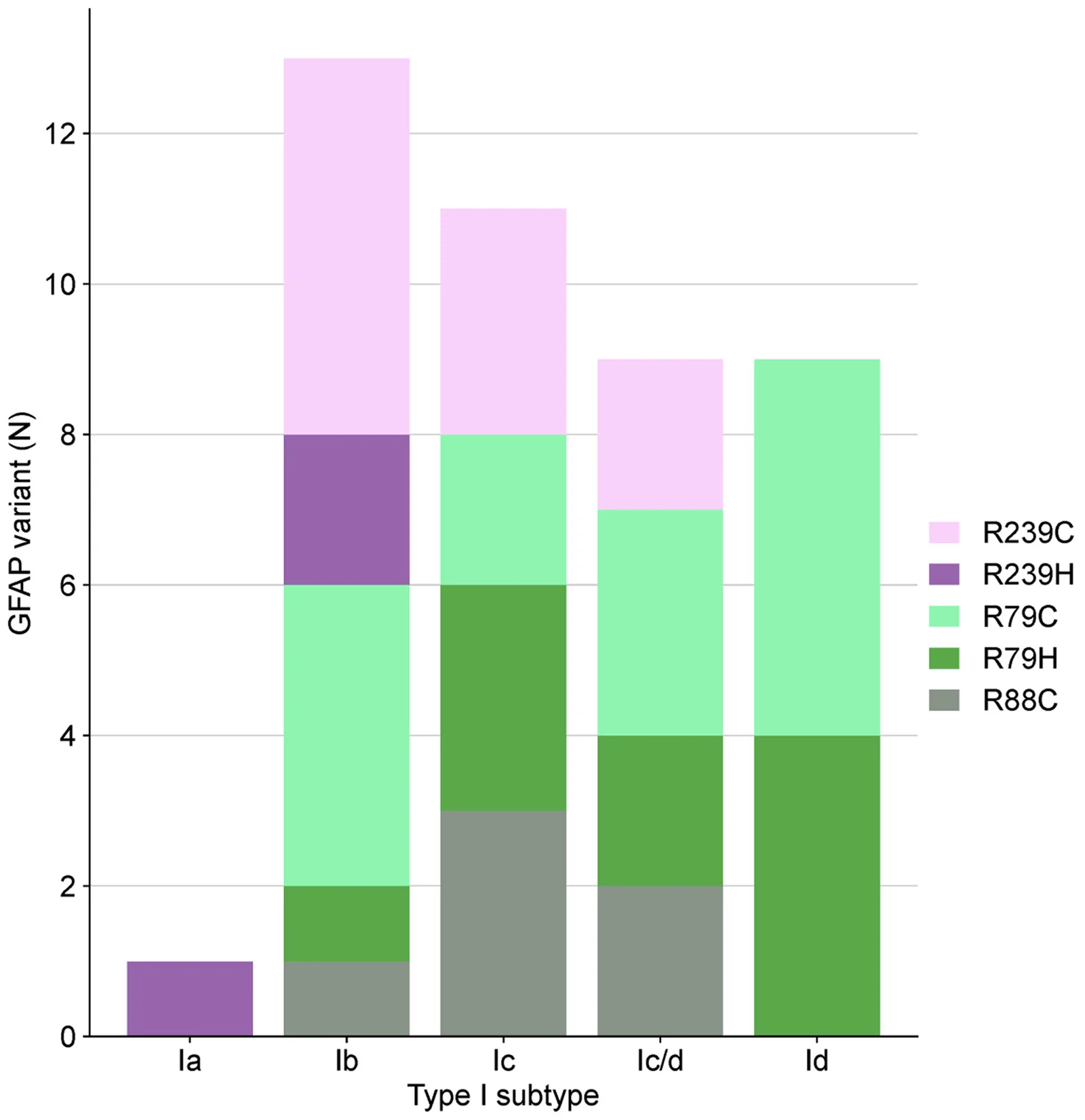
Distribution of the most abundant *GFAP* Arg residues in AxD Type I subtypes. Distribution of Type I AxD subtype across the most frequent variants affecting Arg R239C, R239H, R79C, R79H, and R88C. The number of patients in each Type I subtype carrying Arg mutations is shown. Variants colour scale is shown on the right of the diagram.

**Fig. 5.**
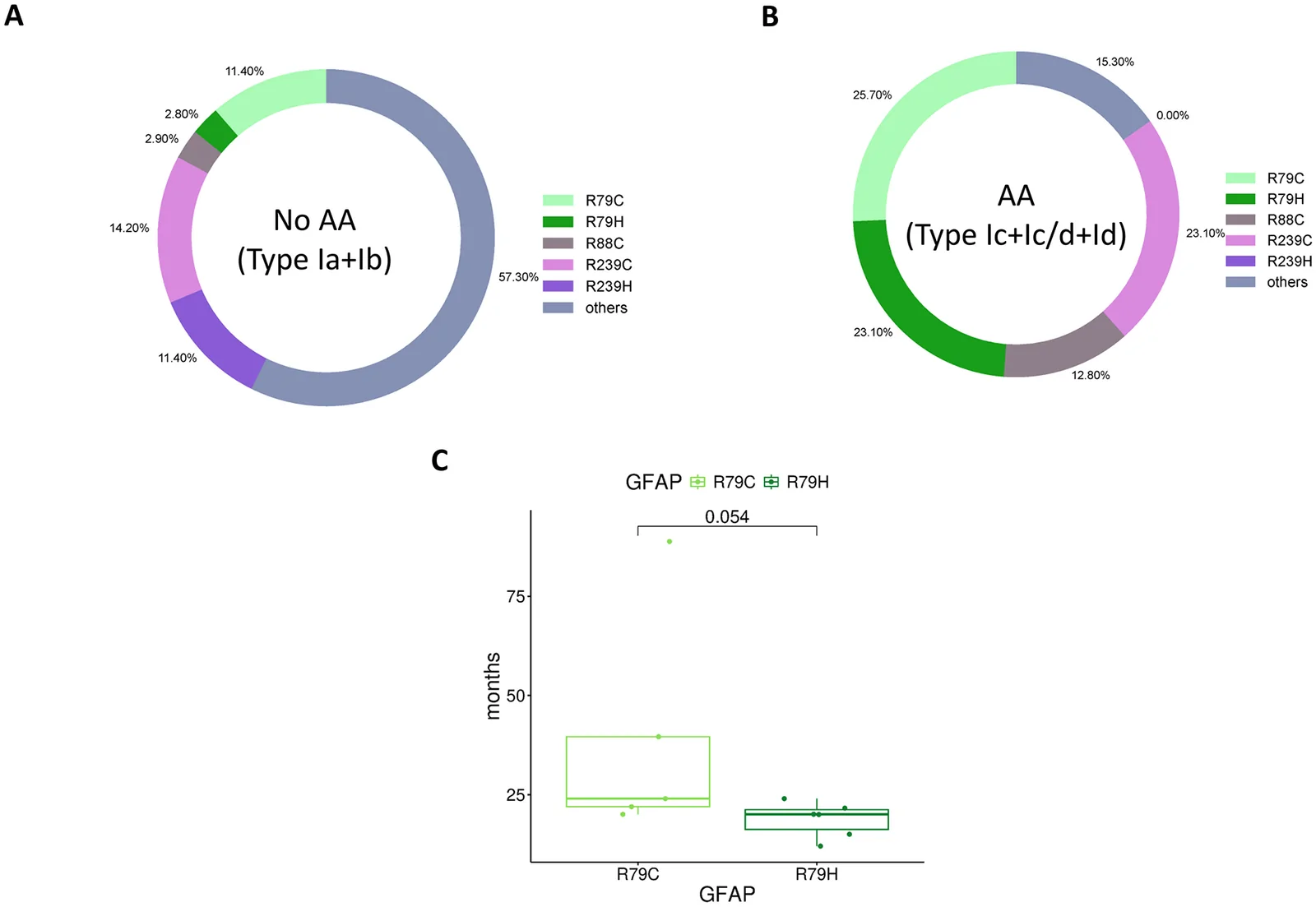
Role of the most frequent Arg variants in autonomous ambulation Contribution in terms of percentage of each R239C, R239H, R79C, R79H, and R88C variant with respect to others mutations in all patients unable (Type Ia + Ib) to acquire autonomous ambulation (AA). Contribution in terms of percentage of each R239C, R239H, R79C, R79H, and R88C variant with respect to others mutations in all patients able to acquire autonomous ambulation (AA) (Type Ic + Ic/d + Id). Box plot shows months of autonomous ambulation (AA) acquisition in patients Ic/d + Id, with no loose of AA yet. Wilcoxon statistical analysis (*p* = 0.05)]

**Fig. 6.**
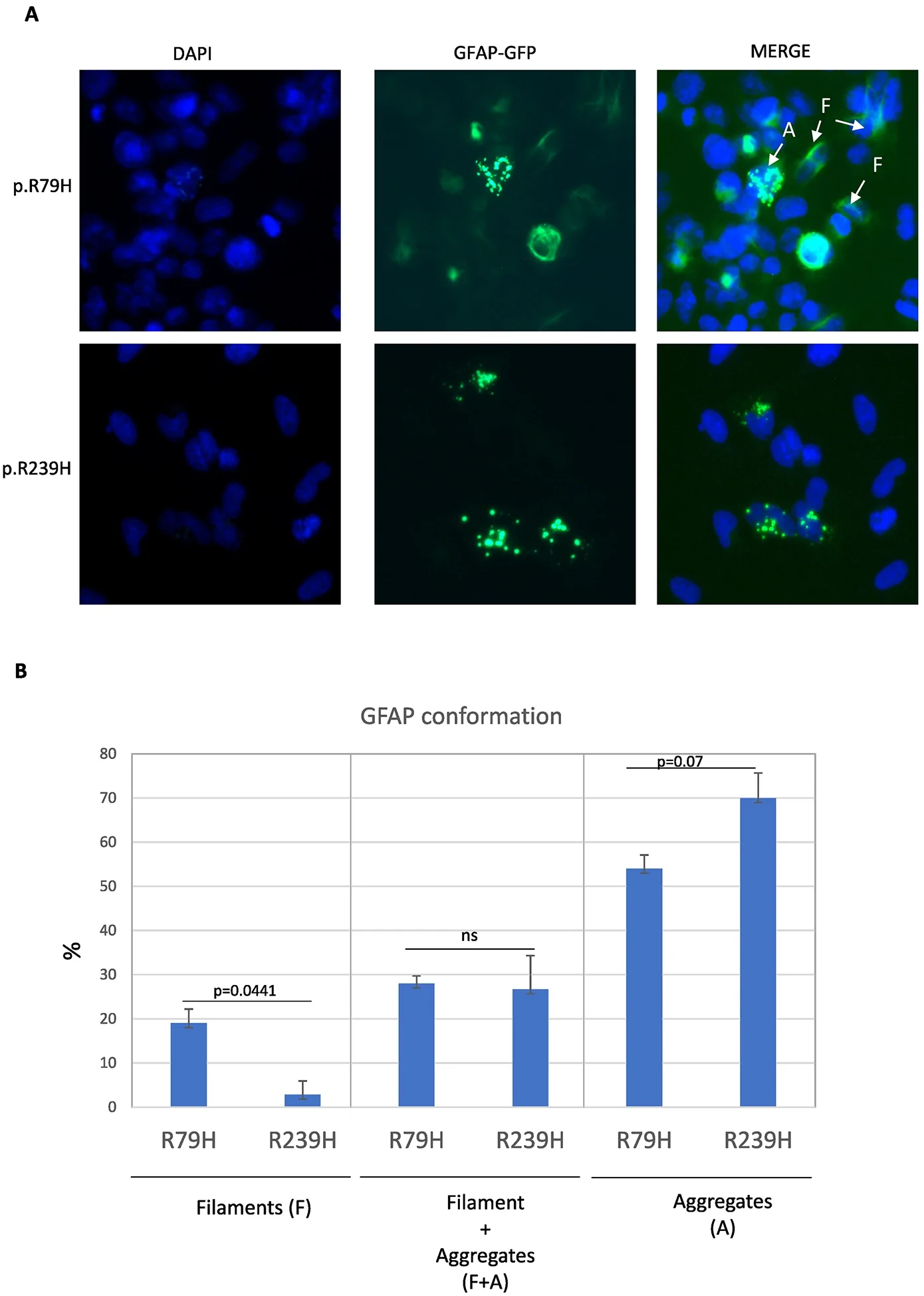
Analysis of p.R79H and p.R239H effects on GFAP conformation Fluorescence microscope images of U251-MG cells transfected with expression plasmids encoding mutant GFAP p.R79H and p.R239H fused to GFP. From left to right, nuclei counterstained with DAPI, GFAP assembly shown as GFP, and merged images. “F” and “A” white arrows indicate patterns showing exclusively GFAP assembling in filaments or aggregates, respectively. Bar diagrams show the different distribution of p.R79H and p.R239H mutant GFAP proteins: only aggregates (A), only filaments (F), mixed (F + A). Paired Student t-test values are indicated for each comparison.

**Fig. 7.**
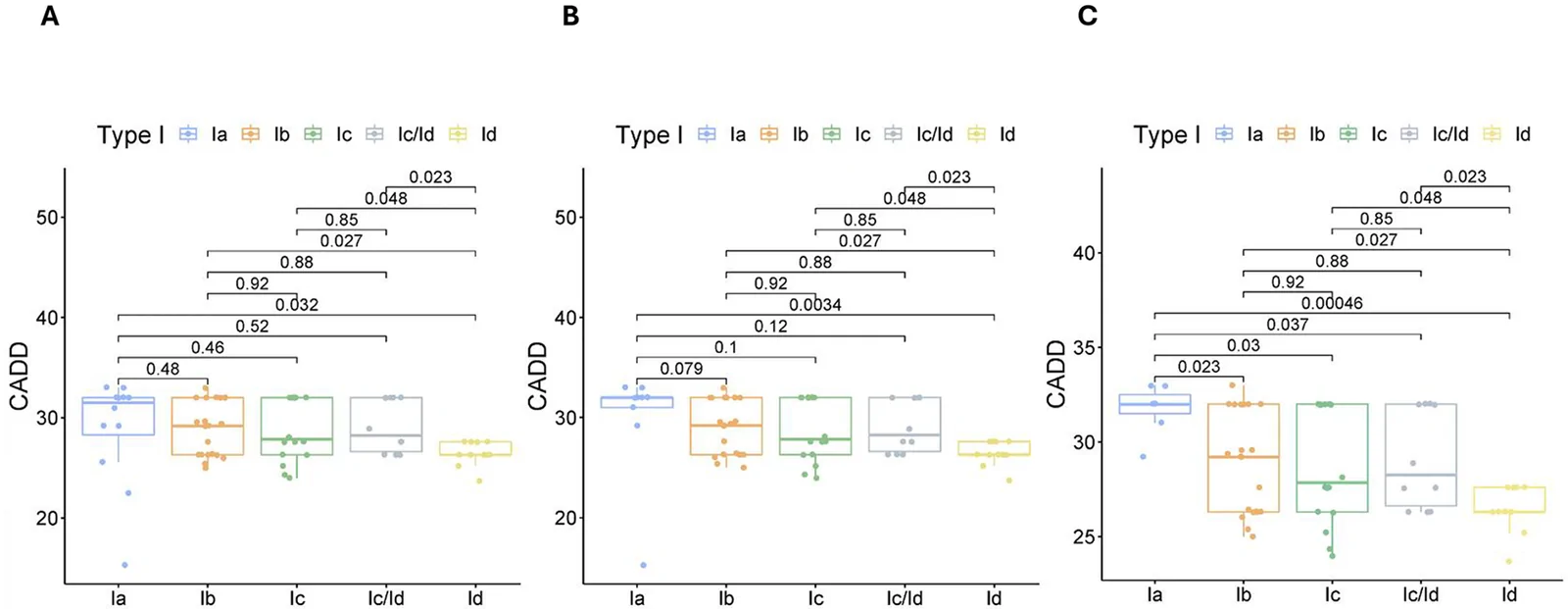
Distribution of CADD score values in AxD Type I subtypes. The box plot shows the distribution of CADD values associated with all GFAP variants. Statistical analysis by Wilcox-test is indicated for two-group comparisons with p-value<0.05 considered as significant. Kruskall-Wallis multi-groups comparison *p* = 0.1. The box plot shows the distribution of CADD values associated with all *GFAP* variants, after the exclusion of p.T396I and p.N77S, predicted to alter the phosphorylation status of GFAP. Statistical analysis by Wilcox-test is indicated for two-group comparisons with p-value<0.05 considered as significant. Kruskall-Wallis multi-groups comparison *p* = 0.02. The box plot shows the distribution of CADD values associated with GFAP variants carried by AxD Type I patients, after the exclusion of phosphor-variants and inherited variants (p.V115F and p.V115I). Statistical analysis by Wilcox-test is indicated for two-group comparisons with p-value. Kruskall-Wallis multi-groups comparison *p* = 0.0051.

**Fig. 8.**
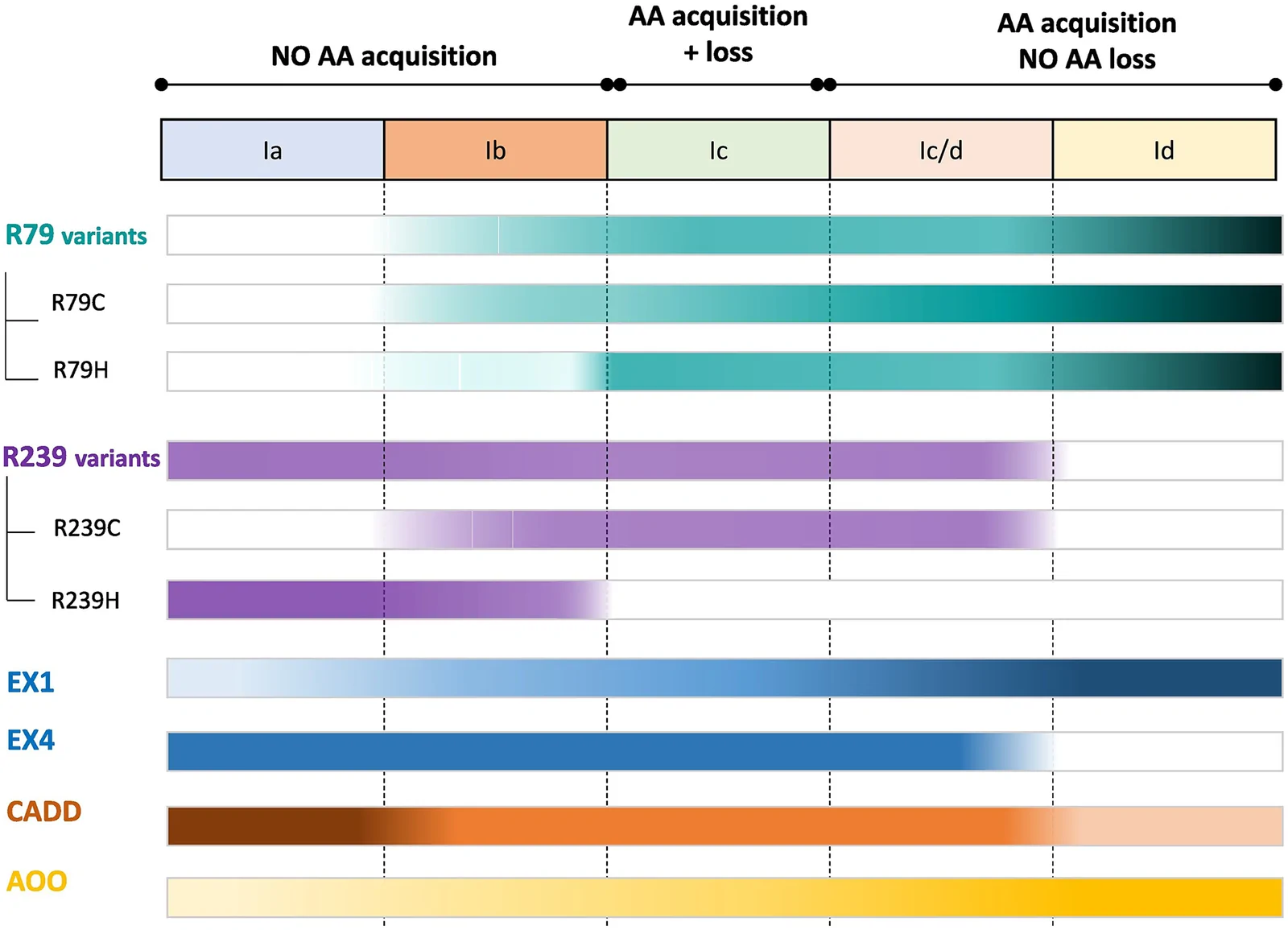
Summary of the variables suitable for prognostic stratification of Type I AxD subtypes. In each subtype progressive increase in colour intensity represents a positive correlation, while a progressive decrease in colour intensity represents a negative correlation. White represents the absence of the variable in the specific subtype. The diagram can be read focusing on the single subtype (Ia, Ib, Ic, Ic/d and Id) or considering intervals based on AA acquisition, as indicated in the upper part of the image. Abbreviations: AA = Autonomous Ambulation, EX = exon, AOO = Age of Onset.

**Table 1:** List of all included patients subdivided by subtypes.

Type of AxD	Amino acid change (HGVS)	EXON	Age at onset (months)	Autonomous ambulation (months)	Loss of autonomous ambulation (years)	literature refences
Ia	T396I	8	0.6	never acquired	N/A	
Ia	N77S	1	2	never acquired		
Ia	R239H	4	0	never acquired		
Ia	E373K	6	1.5	never acquired		Li et al. 2005
Ia	349HLIns	6	1	never acquired		Li et al. 2005
Ia	A364P	6	0	never acquired		Li et al. 2005
Ia	V115F	1	0	never acquired		Li et al. 2005
Ia	L352P	6	0	never acquired		Bassuk et al. 2003
Ia	V115I	1	0	never acquired		Van der Knaap et al. 2005
Ia	R239H	4	0	never acquired		Sakakibara et al. 2007
Ia	F80S	1	3	never acquired		Da Silva Pereira et al. 2013
Ia	L369P	6	0	never acquired		Knuutinen et al. 2018
Ia	Y366C	6	0	never acquired		Takeuchi et al. 2022
Ia	T396I	8	4	never acquired		Papropka et al. 2024
Ib	R239C	4	3	never acquired		
Ib	L76F	1	1.2	never acquired		
Ib	R88C	1	4	never acquired		
Ib	R79C	1	2.04	never acquired		
Ib	R88S	1	0.96	never acquired		
Ib	R79C	1	4.8	never acquired		
Ib	I84F	1	18	never acquired		
Ib	R239C	4	14	never acquired		
Ib	R239C	4	6	never acquired		
Ib	R79C	1	18	never acquired		
Ib	R239C	4	17	never acquired		
Ib	R239C	4	7	never acquired		Meins et al. 2002
Ib	R239P	4	12	never acquired		Meins et al. 2002
Ib	N386I	7	5	never acquired		Cáceres-Marzal et al. 2006
Ib	E333G	6	5	never acquired		Kawai et al. 2006
Ib	Y366C	6	4	never acquired		Hartmann et al. 2007
Ib	R79C	1	9	never acquired		Mignot et al. 2009
Ib	R239H	4	5	never acquired		Ashrafi et al. 2013
Ib	R79H	1	6	never acquired		Ashrafi et al. 2013
Ib	E312K	6	3.5	never acquired		Bonthius et al. 2016
Ib	R239H	4	4	never acquired		Goyal et al. 2014
Ic	V87I	1	16	24	11.5	
Ic	R79L	1	10	13.32	8	
Ic	R416W	8	9	24	11.5	
Ic	R239C	4	24	21.96	6	
Ic	R79C	1	4	21.96	6.8	
Ic	R239C	4	9	20.04	9	
Ic	R88C	1	72	18	11	
Ic	R88C	1	6	12.96	11.17	
Ic	R239C	4	18	24	6.3	
Ic	R79C	1	15	42	6	
Ic	R88C	1	1.33	1.33	11	
Ic	239C	4	35	nda	7	Shiroma et al. 2003
Ic	R79H	1	6	60	7	Brockmann et al. 2004
Ic	R79H	1	6	42	7	Brockmann et al. 2004
Ic	Y83H	1	72	nda	6	Wu et al. 2008
Ic	R79H	1	96	24	7	Paprocka et al. 2019
Ic/Id	R88C	1	7	24	never lost	
Ic/Id	R79C	1	20.04	20.04	never lost	
Ic/Id	R79C	1	15	39.6	never lost	
Ic/Id	L97P	1	14.4	35.4	never lost	
Ic/Id	R239C	4	3.6	30	never lost	
Ic/Id	R88C	1	21.96	24	never lost	
Ic/Id	R79C	1	10	21.96	never lost	
Ic/Id	R239C	4	9	16.8	never lost	
Ic/Id	R79H	1	15	24	never lost	
Ic/Id	R79H	1	60	20	never lost	Asahina et al. 2006
Ic/Id	A364V	6	48	20	never lost	Hartmann et al. 2007
Ic/Id	L359V	6	36	42	never lost	Ramesh et al. 2013
Id	R79H	1	4	21.6	never lost	
Id	R79H	1	18	12	never lost	
Id	A98P	1	18	24	never lost	
Id	V87I	1	24	15.96	never lost	
Id	R79H	1	20	20.04	never lost	
Id	R79C	1	5	24	never lost	
Id	R79C	1	18	88	never lost	
Id	R79H	1	9.6	15	never lost	
Id	R79C	1	6	>20 m	12	Kirsch et al. 2021
Id	R79C	1	18	yes but age not available	never lost	Shiroma et al. 2003
Id	R79C	1	18	yes but age not available	never lost	Shiroma et al. 2003

Red font was used for patients from our cohort; black font for patients abstracted from literature.

N/A=not applicable, NDA=no data available.

## Data Availability

Data will be made available on request.

## Appendix A. Supplementary data

Supplementary data to this article can be found online at https://doi.org/10.1016/j.ymgme.2025.109689.
