# Supplementary material for: Genotype-phenotype correlations of GFAP variants in type I Alexander disease subtypes
Source: Mol Genet Metab. Author manuscript; Available in PMC 2026 Oct 21. (PMC13684854; doi:10.1016/j.ymgme.2025.109689)
Supplement: Supplementary file 1 [file NIHMS2215077-supplement-Supplementary_data.docx]

**SUPPLEMENTAL INFORMATION**

Ia

Ib

Ic

Ic/d

Id

Median AOO (months)

**Legend to Suppl. Figure 1**

Correlation analysis of AOO median values, expressed in months, and AxD Type I subtypes.
Regression coefficient R² =0.9198

Ib

Ic

Ic/d

Ia

Id

%

|  | mediana AOO |
| --- | --- |
| Ia | 0.1 |
| Ib | 5 |
| Ic | 10 |
| Ic/d | 15 |
| Id | 18 |

|  | EX1 |
| --- | --- |
| Ia | 28.5714 |
| Ib | 42.8571 |
| Ic | 56.25 |
| Ic/Id | 66.6667 |
| Id | 100 |

**Legend to Suppl. Figure 2**

Correlation analysis of *GFAP* exon 1 involvement, expressed as percentage (%) of *GFAP* variants lying in exon 1 in each disease subtypes.

Regression coefficient R² =0.9468

Ia

Ic/d

Id

Ib

Ic

Arg > Cys changes (%)

|  | % |
| --- | --- |
| Ia | 0 |
| Ib | 47.5 |
| Ic | 56.2 |
| Ic/Id | 75 |
| Id | 100 |

**Legend to Suppl. Figure 3**

The regression plot shows the correlation between the percentage of variants affecting Arginine (Arg) leading to a Cysteine (Cys) change (Arg>Cys) and milder disease subtypes. R² = 0.9402

**Legend to Suppl. Figure 4**

A. The box plot shows the distribution of CADD values associated with all *GFAP* variants grouping subtypes based on the inability (Ia+Ib) and ability (Ic+Ic/Id+Id) to acquire AA.

B. The box plot shows the distribution of CADD values grouping patients based on AA (Ia+Ib vs Ic+Ic/d+Id), without p.T396I and p.N77S,

predicted to alter the phosphorylation status of GFAP.

C. The box plot shows the distribution of CADD values associated with GFAP, without phosphor-variants and inherited variants p.V115F and p.V115I,

grouping patients based on the inability (Ia+Ib) and ability (Ic+Ic/Id+Id) to acquire autonomous ambulation (AA).

**Suppl. Table 1. Phosphorylation sites in the GFAP protein**

N77S acquisition and T396I loss are indicated on the right of the WT protein
